# Using BCG vaccine to enhance non-specific protection of health care workers during the COVID-19 pandemic: A structured summary of a study protocol for a randomised controlled trial in Denmark

**DOI:** 10.1186/s13063-020-04714-3

**Published:** 2020-09-17

**Authors:** Anne Marie Rosendahl Madsen, Frederik Schaltz-Buchholzer, Thomas Benfield, Morten Bjerregaard-Andersen, Lars Skov Dalgaard, Christine Dam, Sisse Bolm Ditlev, Gulia Faizi, Isik Somuncu Johansen, Poul-Erik Kofoed, Gitte Schultz Kristensen, Ellen Christine Leth Loekkegaard, Christian Backer Mogensen, Libin Mohamed, Anne Ostenfeld, Emilie Sundhaugen Oedegaard, Marcus Kjaer Soerensen, Christian Wejse, Aksel Karl Georg Jensen, Sebastian Nielsen, Tyra Grove Krause, Mihai G. Netea, Peter Aaby, Christine Stabell Benn

**Affiliations:** 1 Bandim Health Project, OPEN, Department of Clinical Research, University of Southern Denmark/Odense University Hospital, Odense, Denmark; 2 Center of Research & Disruption of Infectious Diseases (CREDID), Department of Infectious Diseases, Copenhagen University Hospital, Amager and Hvidovre Hospital, Hvidovre, Denmark; 3 Department of Medicine, Sydvestjysk Hospital, Esbjerg, Denmark; 4 Department of Medicine, Herning Hospital, Herning, Denmark; 5 Department of Respiratory Medicine, Bispebjerg and Frederiksberg Hospital, Copenhagen, Denmark; 6 The Copenhagen Center for Translational Research (CCTR), Bispebjerg Hospital, Copenhagen, Denmark; 7 Department of Infectious Diseases, Odense University Hospital, Odense, Denmark; 8 Department of Pediatrics, Lillebaelt Hospital, Kolding, Denmark; 9 Department of Medicine, Soenderjylland Hospital, Aabenraa, Denmark; 10 Department of Gynaecology and Obstetrics, Nordsjaellands Hospital, Hilleroed, Denmark; 11 Department of Infectious Diseases, Aarhus University Hospital, Skejby, Denmark; 12 Section of Biostatistics, Department of Public Health, University of Copenhagen, Copenhagen, Denmark; 13 Statens Serum Institut, Copenhagen, Denmark; 14 Department of Internal Medicine, Radboud University Medical Centre, Nijmegen, the Netherlands; 15 Department for Genomics & Immunoregulation, Life and Medical Sciences Institute (LIMES), University of Bonn, Bonn, Germany

## Abstract

**Objectives:** The Bacille Calmette-Guérin (BCG) vaccine against tuberculosis is associated with non- specific protective effects against other infections, and significant reductions in all-cause morbidity and mortality have been reported. We aim to test whether BCG vaccination may reduce susceptibility to and/or the severity of COVID-19 and other infectious diseases in health care workers (HCW) and thus prevent work absenteeism.

The primary objective is to reduce absenteeism due to illness among HCW during the COVID-19 pandemic. The secondary objectives are to reduce the number of HCW that are infected with SARS-CoV-2, and to reduce the number of hospital admissions among HCW during the COVID-19 pandemic.

Hypothesis: BCG vaccination of HCW will reduce absenteeism by 20% over a period of 6 months.

**Trial design:** Placebo-controlled, single-blinded, randomised controlled trial, recruiting study participants at several geographic locations. The BCG vaccine is used in this study on a different indication than the one it has been approved for by the Danish Medicines Agency, therefore this is classified as a phase III study.

**Participants:** The trial will recruit 1,500 HCW at Danish hospitals.

To be eligible for participation, a subject must meet the following criteria: Adult (≥18 years); Hospital personnel working at a participating hospital for more than 22 hours per week.

A potential subject who meets any of the following criteria will be excluded from participation in this study:

- Known allergy to components of the BCG vaccine or serious adverse events to prior BCG administration
- Known prior active or latent infection with *Mycobacterium tuberculosis (M. tuberculosis)*
- or other mycobacterial species
- Previous confirmed COVID-19
- Fever (>38 C) within the past 24 hours
- Suspicion of active viral or bacterial infection
- Pregnancy
- Breastfeeding
- Vaccination with other live attenuated vaccine within the last 4 weeks
- Severely immunocompromised subjects. This exclusion category comprises:
- a) subjects with known infection by the human immunodeficiency virus (HIV-1)
- b) subjects with solid organ transplantation
- c) subjects with bone marrow transplantation
- d) subjects under chemotherapy
- e) subjects with primary immunodeficiency
- f) subjects under treatment with any anti-cytokine therapy within the last year
- g) subjects under treatment with oral or intravenous steroids defined as daily doses of 10 mg prednisone or equivalent for longer than 3 months
- h) Active solid or non-solid malignancy or lymphoma within the prior two years
- Direct involvement in the design or the execution of the BCG-DENMARK-COVID trial

**Intervention and comparator:** Participants will be randomised to BCG vaccine (BCG-Denmark, AJ Vaccines, Copenhagen, Denmark) or placebo (saline). An adult dose of 0.1 ml of resuspended BCG vaccine (intervention) or 0.1 ml of sterile 0.9% NaCl solution (control) is administered intradermally in the upper deltoid area of the right arm. All participants will receive one injection at inclusion, and no further treatment of study participants will take place.

**Main outcomes:** Main study endpoint: Days of unplanned absenteeism due to illness within 180 days of randomisation.

Secondary study endpoints: The cumulative incidence of documented COVID-19 and the cumulative incidence of hospital admission for any reason within 180 days of randomisation.

**Randomisation:** Randomisation will be done centrally using the REDCap tool with stratification by hospital, sex and age groups (+/- 45 years of age) in random blocks of 4 and 6. The allocation ratio is 1:1.

**Blinding (masking):** Participants will be blinded to treatment. The participant will be asked to leave the room while the allocated treatment is prepared. Once ready for injection, vaccine and placebo will look similar, and the participant will not be able to tell the difference.

The physicians administering the treatment are not blinded.

**Numbers to be randomised (sample size):** Sample size: N=1,500. The 1,500 participants will be randomised 1:1 to BCG or placebo with 750 participants in each group.

**Trial Status:** Current protocol version 5.1, from July 6, 2020.

Recruitment of study participants started on May 18, 2020 and we anticipate having finished recruiting by the end of December 2020.

**Trial registration:** The trial was registered with EudraCT on April 16, 2020, EudraCT number: 2020-001888-90, and with ClinicalTrials.gov on May 1, 2020, registration number NCT04373291.

**Full protocol:** The full protocol is attached as an additional file, accessible from the Trials

website (Additional file 1). In the interest in expediting dissemination of this material, the familiar formatting has been eliminated; this Letter serves as a summary of the key elements of the full protocol.

**Keywords:** COVID-19, Randomised controlled trial, Protocol, BCG vaccine, NSEs/Non-specific effects of vaccines, Heterologous effects of vaccines, Health care workers, Pandemic, Immune training.

## Supplementary information

**Additional file 1.**

## Data Availability

Trial data is registered and stored via OPEN (Open Patient data Explorative Network, University of Southern Denmark and Odense University Hospital, Denmark) on secure servers in the Region of Southern Denmark. The investigators and sponsors representative will have access to the final dataset. Data will be available from the author on reasonable request and pending ethics approval (arosendahl@health.sdu.dk).

