# Supplementary material for: Using BCG vaccine to enhance non-specific protection of health care workers during the COVID-19 pandemic: A structured summary of a study protocol for a randomised controlled trial in Denmark
Source: Trials. 2020 Sep 17;21:799. doi: 10.1186/s13063-020-04714-3 (PMC7495402; doi:10.1186/s13063-020-04714-3)
Supplement: Supplementary file 1 — Additional file 1. [file 13063_2020_4714_MOESM1_ESM.docx]

**Using BCG vaccine to enhance non-specific protection of health care workers during the COVID-19 pandemic. A randomized controlled multi-center trial.**

**(BCG-DENMARK-COVID)**

Protocol version 5.1, 06-07-2020

EudraCT: 2020-001888-90

# PROTOCOL SIGNATURE SHEET

| **Name** | **Signature** | **Date** |
| --- | --- | --- |
| **Sponsor:**  **Christine Stabell Benn, MD, Prof. University of Southern Denmark Clinical Institute, OPEN**  **Bandim Health Project Studiestræde 6, 1455 Copenhagen K** | 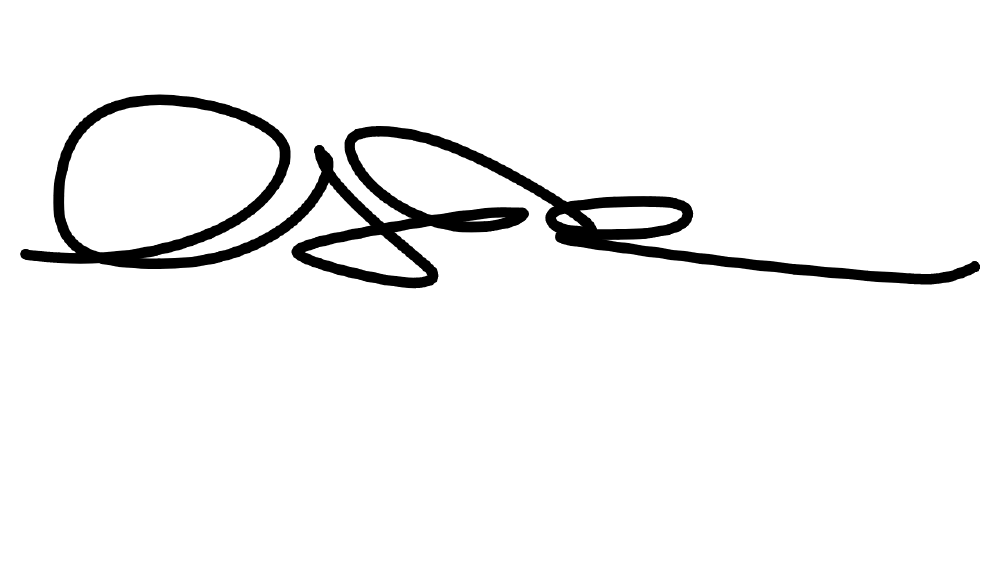 | 6/7-2020 |
| **Coordinating Principal Investigator:**  **Anne Marie Rosendahl Madsen, MD University of Southern Denmark Clinical Institute, OPEN**  **Heden 16, 5000 Odense C** | 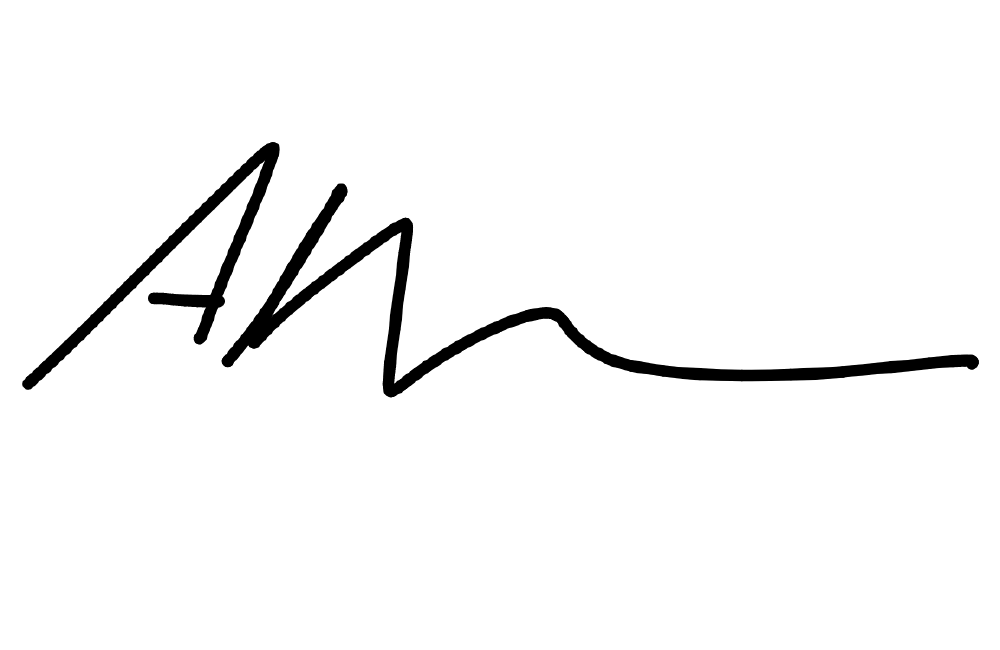 | 6/7-2020 |

| **Protocol title** | **Using BCG vaccine to enhance non-specific protection of health care workers during the COVID-19 pandemic. A randomized controlled**  **multi-center trial.** |
| --- | --- |
| **Protocol ID** | BCG-DENMARK-COVID |
| **Short title** | BCG vaccine for healthcare workers against COVID-19 |
| **EudraCT number** | 2020-001888-90 |
| **Version** | 5.1 |
| **Date** | 06-07-2020 |
| **Sponsor** | Christine Stabell Benn OPEN  Department of Clinical Research,  University of Southern Denmark |
| **Principal Investigator (PI)** | Anne Marie Rosendahl Madsen, MD, PhD student OPEN  Department of Clinical Research,  University of Southern Denmark |
| **Investigator** | Frederik Schaltz-Buchholzer, MD, PhD Fellow Department of Clinical Research,  University of Southern Denmark |
| **Collaborators at participating hospitals:** | |
| **Odense Universityhospital, Odense and Svendborg** | Isik Somuncu Johansen, Dep. of Infectious Diseases, Odense. |
| **Hvidovre/Amager/Glostrup Hospital** | Thomas Lars Benfield, Dep. of Infectious Diseases, Hvidovre. |
| **Aarhus Universitetshospital, Skejby** | Christian Wejse, Dep. of Infectious Diseases, Skejby. |
| **Lillebælt Hospital, Kolding, Vejle** | Poul-Erik Kofoed, Pediatric department, Kolding. |
| **Sønderjylland Hospital, Aabenraa, Sønderborg** | Christian Backer Mogensen, Dep. of Medicine, Aabenraa. |
| **Herning and, Holstebro Hospitals** | Lars Skov Dalgaard, Dep. of Medicine, Herning. |
| **North Zealand Hospital, Hillerød** | Ellen Løkkegaard, Dep. of Gynecology and bstetrics, Hillerød. |
| **Bispebjerg and Frederiksberg Hospital** | Sisse Ditlev, laboratory department, Bispebjerg. |
| **Esbjerg Hospital** | Morten Bjerregaard-Andersen, Dep. of Medicine, Esbjerg. |
|  | |
| **Independent statistician** | Aksel Georg Jensen, University of Copenhagen |
| **Pharmacy** | Hospital Pharmacy of Funen Odense Universitetshospital  Solfaldsvej 38, 5000 Odense C |
| **GCP monitor** | GCP unit  Odense University Hospital  J. B. Winsløws Vej 19, 2. sal 5000 Odense C |
| **Other collaborators:** | |
| **Odense Patient data Explorative Network (OPEN)** | Odense University Hospital, University of Southern Denmark  J. B. Winsloews Vej 9 a, 3  5000 Odense C |
| **Department of Clinical Immunology, Odense University Hospital** | Odense University Hospital  J.B. Winsloews Vej 4,  5000 Odense C |

**LIST OF ABBREVIATIONS AND RELEVANT DEFINITIONS**

| ABR | General Assessment and Registration form (ABR form), the application form that is  required for submission to the accredited Ethics Committee |
| --- | --- |
| AE | Adverse Event |
| AR | Adverse Reaction |
| BCG | Bacillus Calmette-Guérin |
| CA | Competent Authority |
| CCMO  COVID-19 | Central Committee on Research Involving Human Subjects  Coronavirus Disease, i.e. the disease caused by SARS-CoV-2. |
| CV  DKMA | Curriculum Vitae  Danish Medicines Agency |
| EC | The regional Committees on Health Research Ethics for Southern Denmark |
| EU | European Union |
| EudraCT | European drug regulatory affairs Clinical Trials |
| GCP | Good Clinical Practice |
| GDPR | General Data Protection Regulation |
| HCW | Health Care Workers |
| B | Investigator’s Brochure |
| IC | Informed Consent |
| IMP | Investigational Medicinal Product |
| IMPD  PI | Investigational Medicinal Product Dossier Primary Investigator |
| (S)AE | (Serious) Adverse Event |
| SPC | Summary of Product Characteristics |
| Sponsor | The sponsor is the party that commissions the organization or performance of the research, for example a pharmaceutical company, academic hospital, scientific organization or investigator. A party that provides funding for a study but does not  commission it is not regarded as the sponsor but referred to as a subsidizing party. |
| SUSAR | Suspected Unexpected Serious Adverse Reaction |
| WHO | World Health Organization |
| WMO | Medical Research Involving Human Subjects Act |

**TABLE OF CONTENTS**

| **SUMMARY** | **8** |
| --- | --- |
| **BACKGROUND** | **9-11** |
| **Known side effects and risks associated with BCG** | **11** |
| **OBJECTIVES** | **11** |
| **HYPOTHESIS** | **11** |
| **PROJECT GROUP** | **12** |
| **METHODS** | **12-17** |
| **Study design and follow-up** | **12-13** |
| **Study population** | **13** |
| **Inclusion criteria** | **14** |
| **Exclusion criteria** | **14** |
| **TREATMENT OF SUBJECTS** | **14** |
| **STUDY PROCEDURES** | **15-16** |
| **Before participation** | **15** |
| **Day of enrolment** | **15** |
| **Randomization, blinding and treatment allocation** | **15** |
| **Day 0 till end of trial** | **16** |
| **End of trial** | **16** |
| **Non-responders** | **16** |
| **Withdrawal of individual subjects** | **16** |
| **ENDPOINTS AND STUDY PARAMETERS** | **17** |
| **STATISTICAL ANALYSIS** | **17-18** |
| **SAMPLE SIZE CALCULATION** | **18** |
| **Premature termination of the study** | **19** |
| **SAFETY REPORTING** | **19** |
| **Adverse events (AEs)** | **19** |
| **Serious adverse events (SAEs)** | **20-21** |
| **Serious adverse reactions (SARs) and suspected unexpected serious adverse reactions (SUSARs)** | **20-21** |
| **Follow-up of adverse events** | **21** |
| **ETHICAL CONSIDERATIONS** | **21** |
| **Benefits and risks assessment** | **21-22** |
| **Compensation for injury** | **22** |
| **ADMINISTRATIVE ASPECTS, MONITORING AND PUBLICATION** | **23-24** |

| **Monitoring and Quality Assurance** | **23** |
| --- | --- |
| **End of trial** | **23** |
| **Public disclosure and publication policy** | **24** |
| **MAJOR MILESTONES** | **25** |
| **PERSPECTIVES** | **25** |
| **REFERENCES** | **26** |

**SUMMARY**

**Background:** The virus SARS-CoV-2 spreads rapidly throughout the world. The epidemic challenges the available hospital capacity, and this will be augmented by absenteeism of healthcare workers (HCW). HCW are at high risk, currently HCW constitute 20% of all the COVID- 19 cases in Denmark. Strategies to prevent absenteeism of HCW are therefore urgently needed. Bacille Calmette-Guérin (BCG) is a vaccine against tuberculosis, with protective non-specific effects against other infections; significant reductions in morbidity and mortality have been reported, and a plausible immunological mechanism has been identified: “trained innate immunity”. We hypothesize that BCG vaccination can reduce HCW absenteeism during the COVID-19 epidemic.

**Objectives:** Primary objective: To reduce absenteeism among HCW at risk of getting infected and transmitting infection during the COVID-19 epidemic. Secondary objective: To reduce the number of HCW that are infected with SARS-CoV-2 during the COVID-19 epidemic and to reduce the number of hospital admissions amongst HCW during the COVID-19 epidemic.

**Study design:** A placebo-controlled single-center randomized controlled trial.

**Study population:** 1500 HCW (nurses, assistant nurses, assistants, porters, physicians and other staff working at hospitals).

**Intervention:** Participants will be randomized 1:1 to intradermal administration of a standard dose of BCG vaccine or placebo (saline).

**Main study parameters/endpoints:** Primary endpoint: Number of days of (unplanned) absenteeism for any reason. Secondary endpoints: Number of days of (unplanned) absenteeism because of documented COVID infection. Cumulative incidence of hospital admissions.

**Risk for participants and impact:** Based on previous experience and randomized controlled trials in adult and elderly individuals, the risks of BCG vaccination are considered low. The objective of this trial is to evaluate the potential beneficial effects of BCG vaccination through a lower work absenteeism rate of HCW and/or a mitigated clinical course of COVID infection. The primary endpoint and frequent interim analyses facilitate maximum efficiency of the trial, so that results can inform policy making during the ongoing epidemic.

# BACKGROUND

On 30 December 2019, a novel enveloped RNA beta-coronavirus was detected from a patient with pneumonia of unknown etiology in Wuhan, the capital city of Hubei province, China. The pathogen was named the severe acute respiratory syndrome coronavirus-2 (SARS-CoV-2) [1,2]. Since beginning of 2020, SARS-CoV-2 spread rapidly throughout China and the rest of the world, with the first detected case in Denmark on 27 February 2020.

The estimated basic reproduction number (R0) of SARS-CoV-2 is 2.2, i.e. on average, each infected person spreads the infection to an additional two persons. SARS-CoV-2 is being transmitted via droplets during close unprotected contact between people [1]. According to the WHO, as of 8 April 2020, 1,435,091 laboratory-confirmed SARS-CoV-2 cases were established. Health-care workers face an elevated risk of exposure to - and infection with - SARS-CoV-2. This reflects a serious threat to hospital personnel capacity in Denmark as the number of COVID-19 patients that require hospital care may well exceed the capacity of hospital personnel. Even if further mobilization of hospital personnel is possible to some extent, it is imperative to ensure the safety, health and fitness of hospital personnel in order to safeguard continuous patient care. Strategies to prevent SARS-CoV-2 infection or to mitigate its clinical consequences are therefore urgently needed. To date, no curative or protective treatments have been identified yet despite ongoing trials.

**BCG vaccine:** Bacillus Calmette-Guérin (BCG) was developed as a vaccine against tuberculosis, but our group has shown that it can protect against death from other infections, it has what we have called non-specific effects (NSEs) [3]. In clinical studies, BCG vaccination was associated with less child mortality, mainly as a result of reduced neonatal sepsis and respiratory infections [4–6]. In a WHO-commissioned meta-analysis, BCG was associated with 42% (95%CI: 24-55%) lower child mortality [7].

**Non-specific effects (NSEs):** NSEs of BCG are not limited to children. An Indonesian trial with 34 subjects has shown that consecutive BCG vaccination for 3 months reduced the incidence of acute upper tract respiratory infections by 80% (95%CI=22-95%) [8]. It has been recently demonstrated that the non-specific beneficial effects of BCG vaccination are due to epigenetic and metabolic reprogramming of innate immune cells such as myeloid cells and NK cells, leading to an increased antimicrobial activity, a process termed ‘trained immunity’ [9]. Upon stimulation with a pathogen, the innate immune system becomes primed and is able to react faster and more efficiently to a secondary (and non-related) stimulus. In experimental studies, BCG has been shown to protect against not only bacterial and fungal infections, but against viral infections such as influenza as well [10]. Furthermore, among humans receiving yellow fever vaccine virus, those who had received BCG had – compared to placebo treated subjects – lower viral load, and improved anti-viral responses (Figure 1) [11]. The observed effects are proposed to be due to modulation of the human innate immune system through ‘trained immunity’ and are lasting for at least one year [9].


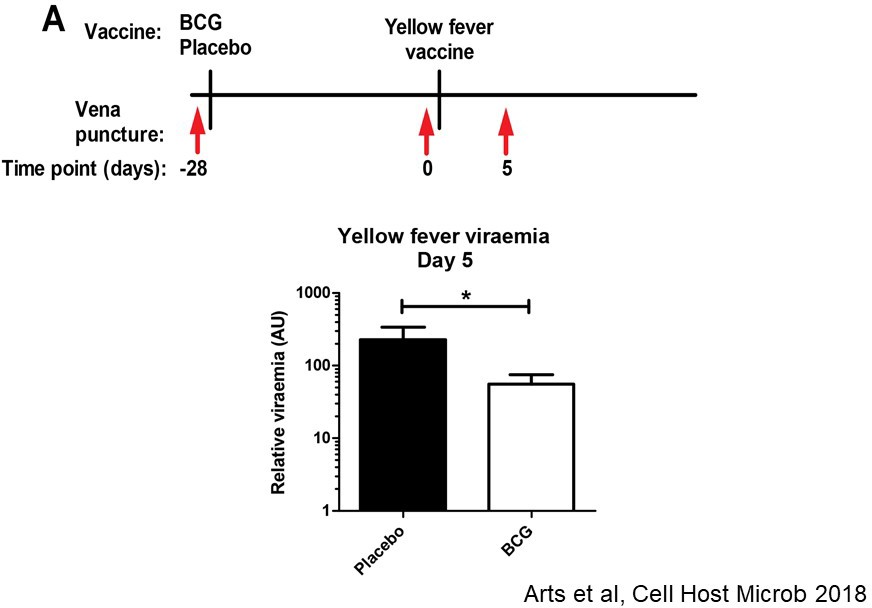
**Figure 1.** Healthy volunteers were injected with either placebo (n=15) or BCG (n=15). One month later all volunteers were injected with yellow fever vaccine. Viremia was assessed on day 5 after yellow fever vaccination by PCR in the blood. BCG vaccination significantly decreased the viremia in the circulation [11].

Based on the capacity of BCG to 1) reduce the incidence of morbidity and mortality due to infectious diseases in children and perhaps in the elderly; 2) exert antiviral effects in experimental models; and 3) reduce viremia in an experimental human model of viral infection, we hypothesize that BCG vaccination may induce (partial) protection against susceptibility to and/or severity of COVID-19. This study will evaluate the efficacy of BCG to improve the clinical course of COVID-19 and to prevent absenteeism.

A randomized controlled trial provides the highest validity for this research question. Given the immediate threat of the COVID-19 epidemic the trial has been designed as a pragmatic study with a highly feasible primary endpoint, that can be continuously measured. This allows for the most rapid identification of a beneficial outcome that would allow other HCW to benefit from the intervention if it is demonstrated to be effective. We have therefore chosen unplanned absenteeism from work as primary outcome, in line with an ongoing clinical trial of BCG in the Netherlands.

# Known side effects and risks associated with BCG: The BCG vaccine has been used for almost 100 years and is now part of more than 100 countries’ childhood immunization programs. In Denmark, we stopped BGC in the 1980s, as the tuberculosis risk decreased. The following side effects are common: Redness and swelling of the skin where the vaccine is given, swollen lymph nodes up to 4 weeks after the vaccine, and a small ~5 mm scar. Because the vaccine has been known and used for many years, we also know the rare side effects: Less than 1 out of 100 can get headaches, fever or wounds above the vaccination site. Less than 1 in 1,000 can get a boil over the vaccination site, and less than 1 in 25,000 can get bone inflammation, lymph node inflammation or severe allergic reaction.

# OBJECTIVES

Primary objective: To reduce absenteeism among HCW during the COVID-19 epidemic. Secondary objective: To reduce the number of HCW that are infected with SARS-CoV-2 during the COVID-19 epidemic and to reduce the number of hospital admissions amongst HCW during the COVID-19 epidemic.

# HYPOTHESIS

BCG vaccination of HCW will reduce absenteeism by 20% over a period of 6 months.

# PROJECT GROUP

Christine Stabell Benn (MD, DMSc), Peter Aaby (DMSc), Anne Marie Rosendahl Madsen (MD, PhD student), Frederik Schaltz-Buchholtzer (MD, PhD student), all from University of Southern Denmark. Mihai Netea (MD, DMSc) Radboud Medical Centre, Nijmegen, The Netherlands. Tyra Groove Krause (MD, PhD) Statens Serum Institut, and the collaborators from each participating hospital.

# METHODS

**Study design and follow-up:** A placebo-controlled adaptive singlecenter randomized controlled trial with inclusion of study participants at several geographic locations. See figure 2 for the logistics of the study. Since the IMP, the BCG vaccine, is used in this study on another indication than the one it has been approved for, this is classified as a phase III study.

**Table 1.** Overview of participating hospitals:

| **Participating hospitals** | **Hospital collaborator** | **Anticipated number of**  **eligible participants**  **(real number enrolled by 26 June)** |
| --- | --- | --- |
| Odense Universitetshospital/  Svendborg Sygehus | Isik Somuncu Johansen | 200 (92) |
| Sygehus Lillebælt, Kolding/Vejle | Poul-Erik Kofoed | 100 (49) |
| Hvidovre/Amager/Glostrup Hospital | Thomas Lars Benfield | 500 (153) |
| Nordsjællands Hospital,  Hillerød | Ellen Løkkegaard | 250 (62) |
| Aarhus Universitetshospital | Christian Wejse | 200 (49) |
| Regionshospitalet Herning/Holstebro | Lars Skov Dalgaard | 100 (69) |
| Sygehus Sønderjylland | Christian Backer Mogensen | 150 (0 – not started yet) |
| Bispebjerg and Frederiksberg Hospital | Sisse Bolm Ditlev |  |
| Esbjerg Sygehus | Morten Bjerregaard-Andersen |  |

**Figure 2:** Study design of the BCG-DENMARK-COVID trial:


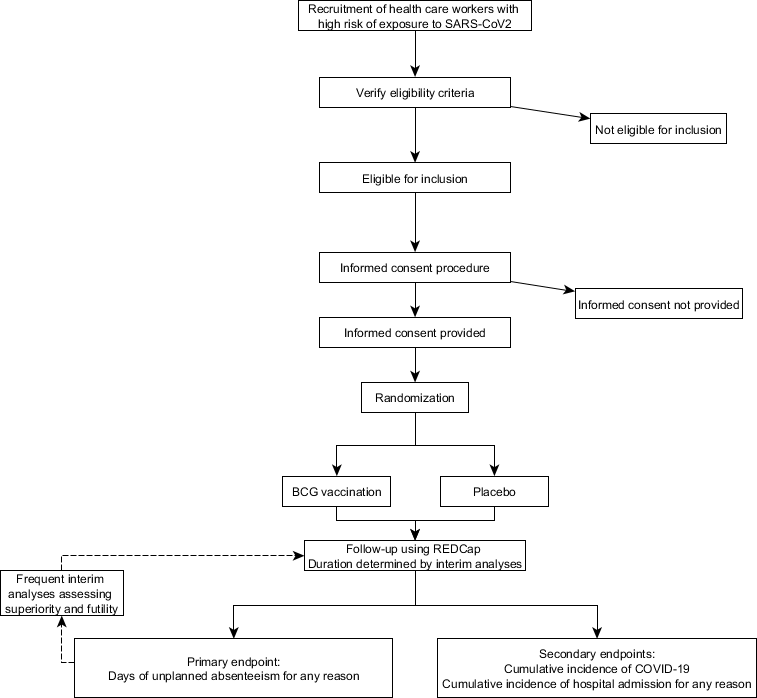


# Study population: As the COVID-19 epidemic has already started in Denmark, our intention is to start as soon as possible. Recruitment of study participants will take place at hospitals where the risk of exposure to SARS-CoV-2 is high. For an overview of participating hospitals, see table 1.

# Inclusion criteria: In order to be eligible to participate in this study, a subject must meet the following criteria: Adult (≥18 years); HCW working at hospitals.

# Exclusion criteria: The exclusion criteria will be assessed at the recruitment interview. We will not use the electronic patient records to check exclusion criteria. A potential subject who meets any of the following criteria will be excluded from participation in this study:

Known allergy to (components of) the BCG vaccine or serious adverse events to prior BCG administration; Known active or latent infection with *Mycobacterium tuberculosis (M. tuberculosis)* or other mycobacterial species; Previous *M. tuberculosis* infection; Previous confirmed COVID-19 infection; Fever (>38 C) within the past 24 hours; Suspicion of active viral or bacterial infection; Pregnancy; Breastfeeding; Vaccination with other live attenuated vaccine within the last 4 weeks; Severely immunocompromised subjects. This exclusion category comprises a) subjects with known infection by the human immunodeficiency virus (HIV-1); b) subjects with solid organ transplantation; c) subjects with bone marrow transplantation; d) subjects under chemotherapy; e) subjects with primary immunodeficiency; f) treatment with any anti-cytokine therapies. g) treatment with oral or intravenous steroids defined as daily doses of 10 mg prednisone or equivalent for longer than 3 months; Active solid or non-solid malignancy or lymphoma within the prior two years; Direct involvement in the design or the execution of the BCG-DENMARK-COVID study; Employed to the hospital < 22 hours per week.

# Treatment of subjects: Participants will be randomized 1:1 to receiving one intradermal BCG vaccine or placebo. Participants that are randomized in the active arm will receive a BCG vaccine (BCG-Denmark, AJ Vaccines, <http://www.produktresume.dk/AppBuilder/login.html>). The vaccine batch provided by AJ Vaccines for this study has not been released for sale in Denmark, but has been released by WHO, certificate number: 2019052989. The product is identical to the vaccine released for Denmark with respect to production, control and contents. AJ Vaccines will send the vaccines to Hospital Pharmacy of Funen. The pharmacy will assure correct control and storage of the vaccines and will release the vaccines to the study according to GCP. A collaboration agreement will be made with the pharmacy. Placebo will be 0.1 ml sterile 0.9 % NaCl, which has a similar color as the resuspended BCG vaccine.

# Description of route of administration and dosage:

All participants will receive one injection at inclusion. No further treatment of study participants will take place. BCG will be administered in the right upper arm, intradermally, 0.1 ml of the suspended vaccine. Placebo will be administered in the right upper arm, intradermally, 0.1 ml of sterile 0.9 % NaCl solution.

**STUDY PROCEDURES**

**Before participation:** HCW (possible subjects) will be informed about the study by their local contact person/hospital collaborator. Also written information about the study will be sent to possible subjects by email. Subjects that are interested will receive additional study information (participant information and informed consent form) and are scheduled for an appointment with one of the investigators. Screening of possible subjects, information, enrolment procedures and follow up will be done by the investigators from University of Southern Denmark or trained MD’s to whom the task has been delegated, at all locations.

The subject visits the vaccination site (a designated room in the hospital), where he/she receives oral and written information by the investigator. Eligibility criteria will be checked. For all women of childbearing potential, pregnancy will be excluded with a urine HCG test according to CTFG’s ’Recommendations related to contraception and pregnancy testing in clinical trials’. Women of childbearing potential are defined in this study as all women above 18 years of age (according to the inclusion criteria), who has not reached menopause. Women will be asked about this at the inclusion interview. Before informed consent is be obtained, subjects are offered 24 h of reflection time.

See figure 3 below for flowchart of interventions.

# Day of enrolment (Day 0): Day 0: Participants fill in a short questionnaire regarding baseline characteristics. The entire follow-up will be performed electronically using REDCap. An email with a link to the questionnaire will be sent to the participants weekly. At enrolment participants will be informed about this procedure. A blood sample of 5 ml will be drawn for subsequent testing for SARS-CoV-2 antibodies.

# Randomization, blinding and treatment allocation: Participants will be randomized to BCG or placebo in a 1:1 ratio. Randomization will be done centrally using the REDCap tool with stratification per hospital and by sex and age groups (+/- 45 years of age) in randomly selected block sizes of 4 and 6.

The BCG vaccine will be administered to participants at all sites by the investigators, who have been trained in intradermal injections. Participants will be blinded to treatment. The participant will be asked to leave the room while the vaccine/placebo is prepared. Once ready for injection, the vaccine and placebo will look similar, and the participant will not be able to tell the difference. The physicians administering the BCG vaccine or placebo will not be blinded. In case of serious adverse events, the participant can be unblinded after consultation with the PI or sponsor. The end of the trial is defined as whichever comes latest: the last patient’s last registration in the online data collection, or 180 days. When the study is ended, all participants receive an email with information about the intervention that they have received (BCG/placebo).

**Day 0 till end of trial (weekly):** Short electronic questionnaire regarding work absenteeism, symptoms and side effects. Link to the questionnaire will be sent every week by email to the participants through REDCap. We will use the participants official work email or e-boks for security reasons. In case a participant reports acute respiratory symptoms indicating COVID-19, he or she will be tested depending on test availability.

**End of trial:** Participants are asked to fill in a final questionnaire. A blood sample of 5 ml will be drawn for subsequent testing for SARS-CoV-2 antibodies.

**Figure 3:** Flowchart, interventions.


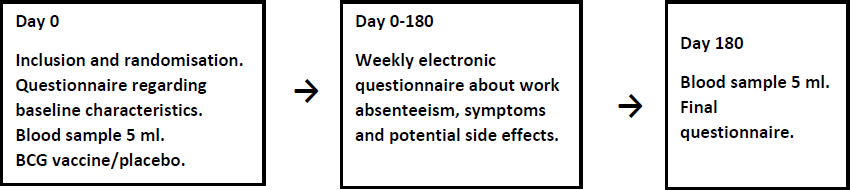


# Non-responders: Day 0 till end of study: In case of not filling in the questionnaire for ≥10 days, the participant receives a notification. Participants with incomplete follow-up despite notification will be telephoned with the request to complete the follow-up data. In case the participant does not answer the telephone, information on the participant will be retrieved from the Danish National Discharge Register regarding hospital admission, ICU admission and outcomes. An explicit permission for this procedure will be requested from the participant at informed consent.

# Withdrawal of individual subjects: Subjects can leave the study at any time for any reason if they wish to do so, without any consequences. The investigator can decide to withdraw a subject from the study for urgent medical reasons. A participant will only be replaced in case of withdrawal before the administration of BCG vaccine/placebo. The participant will be asked to give a blood sample for SARS-CoV-2 antibody testing at end of the study period – even if they for some reason leave the study prematurely.

**ENDPOINTS**

**Main study endpoint:** Number of days of unplanned absenteeism for any reason.

# Secondary study endpoints: The cumulative incidence of documented COVID and the cumulative incidence of hospital admission for any reason.

# Furthermore, in line with the Dutch trial, the following endpoints will be assessed:

The number of days of unplanned absenteeism, because of documented SARS-CoV-2 infection; The number of days of absenteeism, because of imposed quarantine as a result of exposure to SARS- CoV-2; The number of days of absenteeism, because of imposed quarantine as a result of having acute respiratory symptoms, fever or documented SARS- CoV-2 infection; The number of days of unplanned absenteeism because of self-reported acute respiratory symptoms; The number of days of self-reported fever (≥38 °C); The number of days of self-reported acute respiratory symptoms; The cumulative incidence of self-reported acute respiratory symptoms; The cumulative incidence of death for any reason; The cumulative incidence of death due to documented SARS- CoV-2 infection; The cumulative incidence of Intensive Care Admission for any reason; The cumulative incidence of Intensive Care Admission due to documented SARS- CoV-2 infection; The cumulative incidence of Hospital Admission due to documented SARS-CoV-2 infection.

# STATISTICAL ANALYSIS

Data will be reported quantitatively. All analyses will be performed from the intention-to-treat principle. Missing data will be dealt with by multiple imputation using the mice package in R.

**Primary study parameter:** The primary endpoint, work absenteeism for any reason, will be reported as the average proportion of sick days with standard deviation. It will be analyzed as counts per week (i.e. multiple observations per subject) using a Bayesian negative binomial regression with a random intercept per participant and a fixed effect for BCG, hospital, observation week, and number of average workdays per week. The brm function from the R package “brms” will be used for fitting the negative binomial model. The effect will be reported as a relative risk with 95% confidence interval (CI). Please refer to section interim analysis for type-1 error control and power.

# Secondary study parameters: Similar regression models will be used for secondary endpoints: Number of days of unplanned absenteeism because of documented SARS-CoV-2 infection; number of days of self-reported fever: negative binomial model, similar to the primary endpoint.

Documented infection with SARS-CoV-2, death for any reason, death due to documented SARS-CoV-2 infection, cumulative incidence of Intensive Care Admission for any reason, cumulative incidence of Intensive Care Admission due to documented SARS-CoV-2 infection, cumulative incidence of Hospital Admission for any reason, cumulative incidence of Hospital Admission due to documented SARS-CoV-2 infection: a Cox proportional hazards model will be used for these endpoints. When applicable (i.e. in the event that one or more participants have died during the follow-up period) a competing events analysis will be performed in addition (Fine & Gray model).

# Other study parameters: Continuous baseline characteristics will be reported as mean and standard deviation or median and inter-quartile range, as appropriate. Categorical baseline characteristics will be reported as count and percentage. No statistical testing for baseline characteristics will be performed.

# Interim analysis: Every month, an interim analysis will be conducted by the independent statistician of the trial. The primary endpoint will be analyzed as described above. The Bayesian model yields a posterior distribution of the relative risk. The posterior probability of the superiority hypothesis (RR < 1) will be calculated as well as the posterior probability of the futility hypothesis (RR > 0.7). If during any of the interim analyses, the posterior probability of superiority is > 0.995 or the posterior probability of futility is > 0.99, a conclusion is reached, and the trial will be stopped. These posterior probability breakpoints have been chosen such that the type-1 error rate is <0.025 (similar to a two-sided alpha of 0.05) and the power of detecting superiority is >90% if the true RR is 0.7.

Monthly, the following secondary endpoints will be reported to the steering group unblinded: ‘the cumulative incidence of hospital admission, Intensive Care Admission or death’.

# SAMPLE SIZE CALCULATION

Interim analyses are done by an independent statistician every month to monitor effects on the primary outcome: days of sickness absence. If the posterior probability of a positive effect (relative risk (RR) <1) exceeds 0.995, the study will be discontinued, and the conclusion reported to the authorities as soon as possible. The parallel Dutch study and participation in this project group by Prof. Netea from The Netherlands will enable combined analysis that will strengthen the conclusions and increase the power to analyze the secondary outcomes and more rare outcomes as intensive hospitalization. As this is an adaptive sample size, the final sample size and follow-up duration is subject to the percentage of absenteeism during the study period, variation in absenteeism between participants, and the true effect of BCG vaccine.

# PREMATURE TERMINATION OF THE STUDY

Based on the interim analyses, the Steering group consisting of the sponsor, the PI and the collaborators from the seven participating hospitals can recommend terminating the trial prematurely. The final decision is with the sponsor.

# SAFETY REPORTING

**Temporary halt for reasons of subject safety:** In accordance to section 10, subsection 4, of the Medical Research Involving Human Subjects Act (WMO), the sponsor will suspend the study if there is sufficient ground that continuation of the study will jeopardize subject health or safety. The sponsor will notify the EC and the DKMA without undue delay of a temporary halt including the reason for such an action. The study will be suspended pending a further positive decision by the EC and the DKMA. The investigator will take care that all subjects are kept informed.

# Adverse events (AEs): Adverse events are defined as any undesirable experience occurring to a subject during the study, whether or not considered related to the investigational product, the placebo or the trial procedures. Participants can fill in adverse events in REDCap using the online application. All adverse events will be reported to the relevant authorities for 7 days after inclusion.

# Serious adverse events (SAEs): A serious adverse event is any untoward medical occurrence or effect that:

- Results in death;
- Is life threatening (at the time of the event);
- Requires hospitalization or prolongation of existing inpatients’ hospitalization;
- Results in persistent or significant disability or incapacity;
- Is a congenital anomaly or birth defect;

Or any other important medical event due to the intervention based upon appropriate judgement by the investigator. An elective hospital admission will not be considered as a serious adverse event. Participants will be asked about the occurrence of SAE’s weekly in the questionnaire. In case of a SAE, dependent on the symptoms of the participant, he/she will be contacted by the investigator and if necessary, admitted to the hospital. The condition of the participant will be evaluated by the investigator, who can decide to un-blind if deemed necessary. In case a participant does not fill in the online questionnaire, he or she will be called by the investigator to collect data on SAE’s.

The investigator will notify sponsor within 24 hours after first knowledge of any SAEs, in order for sponsor to assess whether it is a SUSAR.

**Serious adverse reactions (SARs) and suspected unexpected serious adverse reactions (SUSARs):** Adverse reactions are all untoward and unintended responses to an investigational product related to any dose administered. The BCG vaccine SPC will be used as reference. Unexpected adverse reactions are SUSARs if the following three conditions are met:

- The event must be serious (see above);
- There must be a certain degree of probability that the event is a harmful and an undesirable reaction to the medicinal product under investigation, regardless of the administered dose;
- The adverse reaction must be unexpected, that is to say, the nature and severity of the adverse reaction is not in agreement with the product information as recorded in the SPC.

The investigator and the sponsor will report expedited the following SUSARs to the EC and the DKMA with consideration of the following timeframes; Within 7 days of first knowledge for SUSARs that result in death or require admission to an intensive care unit followed by a period of maximum of 8 days to complete the initial preliminary report. All other SUSARs will be reported within a period of maximum 15 days after the sponsor has first knowledge of the serious adverse events. Reporting will be done using the *eBlanket* provided by the DKMA.

- SUSARs that have arisen in the clinical trial that was assessed by the EC and DKMA;
- SUSARs that have arisen in other clinical trials of the same sponsor and with the same medicinal product, and that could have consequences for the safety of the subjects involved in the clinical trial that was assessed by the EC and DKMA.

The remaining SUSARs are recorded in an overview list (line-listing) that will be submitted to the EC at the end of trail. This line-listing provides an overview of all SUSARs from the study medicine, accompanied by a brief report highlighting the main points of concern. There will be no yearly report as the study will be completed before one year.

The expedited reporting will occur not later than 15 days after the sponsor has first knowledge of the adverse reactions. For fatal or life-threatening cases, the term will be maximal 7 days for a preliminary report with another 8 days for completion of the report.

# Follow-up of adverse events: All AEs occurring in the first 7 days after inclusion and all SAEs, SARs and SUSARs will be followed until they have abated, or until a stable situation has been reached. Depending on the event, follow up may require additional tests or medical procedures as indicated, and/or referral to the general physician or a medical specialist.

AEs will only be reported during the first 7 days after inclusion. SUSARs will be reported to the EC and DKMA till the end of the study.

# ETHICAL CONSIDERATIONS

The study will be conducted according to the principles of the Declaration of Helsinki amended at the General Assembly in October 2013 and in accordance with the Medical Research Involving Human Subjects Act (WMO) and other guidelines, regulations and Acts. The study will be submitted for approval from the EC and DKMA. We will ask participants permission to access electronic patient records, in order to access relevant test results and information on hospital admissions in the study period. We only need to access the records **after** the participants have given their consent. The participants will be informed, that their consent gives the sponsor, sponsors representatives, PIs, and monitoring agencies direct access to relevant health information from their electronic patient records.

# Benefits and risks assessment: The potential benefit for subjects randomized to the BCG-arm is possibly a lower risk of getting COVID-19 and a lower risk of severe illness related to COVID-19, which could reduce absenteeism and the risk of hospital admission. Potential risks include only the well-known side effects of the vaccine. The BCG vaccine SPC will be used as reference document. As part of the study information, participants will be informed about the possible side effects to the treatment, including that it is common and not treatment demanding that, following BCG vaccination, redness, swelling and ulceration of the skin where the vaccine is given will occur, that there may be swollen lymph nodes in the area some weeks after the vaccine is given, and that in the longer term, a small scar, approximately 5 mm diameter, will appear where the vaccine was given. This kind of side effects will not be reported to DKMA.

Severe but very rare side effects are injection site abscesses, BCG lymphadenitis, disseminated BCG disease, osteitis, osteomyelitis, anaphylaxis, formation of keloid/lupoid, suppurative lymphadenitis. A complete list of known side effects can be found in the participant information leaflet. Subjects that receive the placebo hardly have any potential risks and no benefits. Local hematoma formation can occur at the site of injection.

BCG vaccine in immunocompetent adult people is considered safe, also for persons with prior BCG vaccination and even in latently infected adults [12,13]. In a randomized controlled trial that compared revaccination with BCG versus placebo, no vaccine-related serious adverse events were observed in the 312 patients in the BCG arm [14].

Thus, the risk to and burden for the subject of BCG vaccination is estimated to be low, according to previous trials that have been performed with BCG vaccines [8,11,14]. It is not recommended to get other live attenuated vaccines within a month of BCG vaccination, unless given at the same time. Apart from this, no interactions are known between BCG vaccination and other medications.

Using an adaptive design, the study aims to find a positive effect of BCG-vaccine on a population level, which could be applied quickly in participants allocated to placebo and implemented to hospitals that do not participate in the study.

Participation in this study should in principle not be an exclusion criterion for participation in other studies (for example into treatments for Coronavirus infections). Participants are informed that the Mantoux test for screening for tuberculosis is no longer reliable after BCG vaccination. The reliability of the TB-Quantiferon test is not affected by BCG-vaccination.

# Compensation for injury: The study participants are fully insured according to the Danish Patient Insurance Act. This insurance provides cover for damage to research subjects through injury or death caused by the study. Participants will not be compensated for participating in this study. Damage to subjects through injury or death, caused by the study or negligence of local study investigators, is not accountable to the principle investigator.

# ADMINISTRATIVE ASPECTS, MONITORING AND PUBLICATION

Data and biological material will be registered and stored via OPEN (Open Patient data Explorative Network) using REDCap, OPEN Analyze and OPEN Biobank hosted by OPEN on secure servers in the Region of Southern Denmark. Data analysis will be done according to safety regulations with pseudonymized copies of data. Data will be processed and stored locked / inaccessible and safely in full compliance with the Data Inspectorate's Standard Terms for Research Projects. All source data will be kept in the electronic CRF in REDCap. The sponsor, investigators appointed by the sponsor, the study statistician, and monitoring agencies will be able to get direct access to source data. A subject identification code list based on non-traceable items will be used to link the data to the subject. The key to the code will be safeguarded by the coordinating PI on a designated secure location.

The handling of personal data complies with the EU General Data Protection Regulation and the Danish Act on Implementation of the General Data Protection Regulation.

The investigator and master file and the electronic data from the eCRF will be stored for a duration of 5 years. Information, data, and results that originate from this study may not be disclosed without the written permission of the sponsor and coordinating principal investigator. The blood samples will be kept in an OPEN research biobank during the study period and potentially remaining biological material will be stored in an OPEN biobank for 5 years after end of trial, allowing for the possibility that new insights may be reached during this time, which could be addressed in the existing material. If that is the case, ethical permission to analyze the samples will be applied for. After 5 years all remaining biological material will be destroyed.

# Monitoring and Quality Assurance: We classify this trial as a low risk study, because the intervention is already registered, and has been used in previous trials. The study will be monitored by the GCP unit at Odense University Hospital. The first monitor visit will take place before inclusion of the first study participant. During this visit the presence and completeness of the relevant Study Files will be checked.

# End of trial: The sponsor will notify the EC and DKMA about the end of the study within a period of 90 days. The end of the study is defined as the last patient’s last registration in the online data capture system. Within one year after the end of the study, the sponsor will submit a final study report with the results of the study, including any publications/abstracts of the study, to the EC.

The sponsor will notify the EC and DKMA immediately of a temporary halt of the study, including the reason of such an action. In case the study is ended prematurely, the sponsor will notify the EC and DKMA within 15 days, including the reasons for the premature termination.

# Public disclosure and publication policy: The results of this study will be disclosed unreservedly at the end of the study. Results that are important for public health will be notified to the competent authorities as soon as possible. The trial will be registered in a public trial registry before the first patient is recruited.

# FUNDING AND MATERIAL SUPPORT

We are currently applying for funding from various sources that support projects aiming to mitigate the effect of the COVID-19 pandemic. AJ Vaccines will be supplying the BCG vaccines free of charge. This company has had no influence on the design of the study and will not have influence on any aspect of analyzing, interpretation or reporting of results. None of the parties in the study group have any economic interest in this company. The EC will be informed about any further economic or other support that the project may receive. This will include the amount received, how it will be used and account number.

# MAJOR MILESTONES

Application for Regional ethical committee submitted: April 16, 2020

Application for EudraCT submitted: April 16, 2020

Application for Danish Medicines Agency submitted: April 16, 2020

Approval from Danish Medicines Agency received: April 27, 2020.

Approval from Regional Ethics Committee received: April 30, 2020.

Inclusion of first participant: May 2020

Inclusion of last participant: December 2020

End of follow-up, last patient: June 2021

Data analyzed: August 2021

# PERSPECTIVES

In this trial the registered BCG vaccine will be used in hospital personnel as protection for absenteeism during the COVID pandemic. The risk of the BCG vaccine is low and only minor local side effects (redness, local pain) are common. The benefits of BCG vaccination based on earlier clinical studies are expected to be a reduction in morbidity and absenteeism of at least 20% compared to unvaccinated individuals, and potentially higher. These effects are mediated by the capacity of BCG to induce trained immunity and may represent an important tool for protection against SARS-CoV-2 virus infection until a specific effective vaccine is developed.

# REFERENCES

1. Novel Coronavirus (2019-nCoV) situation reports. https://[www.who.int/emergencies/diseases/novel-coronavirus-2019/situation-reports](http://www.who.int/emergencies/diseases/novel-coronavirus-2019/situation-reports) (accessed 15 Apr 2020).
2. Huang C, Wang Y, Li X, *et al.* Clinical features of patients infected with 2019 novel coronavirus in Wuhan, China. *The Lancet* 2020;**395**:497–506. doi:10.1016/S0140-6736(20)30183-5
3. Aaby P, Kollmann TR, Benn CS. Nonspecific effects of neonatal and infant vaccination: public- health, immunological and conceptual challenges. *Nat Immunol* 2014;**15**:895–9. doi:10.1038/ni.2961
4. Biering-Sørensen S, Aaby P, Napirna BM, *et al.* Small randomized trial among low-birth-weight children receiving bacillus Calmette-Guérin vaccination at first health center contact. *Pediatr Infect Dis J* 2012;**31**:306–8. doi:10.1097/INF.0b013e3182458289
5. Kristensen I, Aaby P, Jensen H. Routine vaccinations and child survival: follow up study in Guinea-Bissau, West Africa. *BMJ* 2000;**321**:1435–8.
6. Aaby P, Roth A, Ravn H, *et al.* Randomized trial of BCG vaccination at birth to low-birth-weight children: beneficial nonspecific effects in the neonatal period? *J Infect Dis* 2011;**204**:245–52. doi:10.1093/infdis/jir240
7. Higgins JPT, Soares-Weiser K, López-López JA, *et al.* Association of BCG, DTP, and measles containing vaccines with childhood mortality: systematic review. *BMJ* 2016;**355**:i5170.
8. Wardhana null, Datau EA, Sultana A, *et al.* The efficacy of Bacillus Calmette-Guerin vaccinations for the prevention of acute upper respiratory tract infection in the elderly. *Acta Medica Indones* 2011;**43**:185–90.
9. Netea MG, Joosten LAB, Latz E, *et al.* Trained immunity: A program of innate immune memory in health and disease. *Science* 2016;**352**:aaf1098. doi:10.1126/science.aaf1098
10. Spencer JC, Ganguly R, Waldman RH. Nonspecific Protection of Mice against Influenza Virus Infection by Local or Systemic Immunization with Bacille Calmette-Guerin. *J Infect Dis* 1977;**136**:171–5. doi:10.1093/infdis/136.2.171
11. Arts RJW, Moorlag SJCFM, Novakovic B, *et al.* BCG Vaccination Protects against Experimental Viral Infection in Humans through the Induction of Cytokines Associated with Trained Immunity. *Cell Host Microbe* 2018;**23**:89-100.e5. doi:10.1016/j.chom.2017.12.010
12. Expert committee on biological standardization: Recommendations to assure the quality, safety and efficacy of BCG vaccines. *WHO Tech Rep Ser* 2011.
13. Hatherill M, Geldenhuys H, Pienaar B, *et al.* Safety and reactogenicity of BCG revaccination with isoniazid pretreatment in TST positive adults. *Vaccine* 2014;**32**:3982–8. doi:10.1016/j.vaccine.2014.04.084
14. Nemes *et al*. Prevention of M. tuberculosis Infection with H4:IC31 Vaccine or BCG Revaccination. NEJM. https://[www.nejm.org/doi/full/10.1056/NEJMoa1714021](http://www.nejm.org/doi/full/10.1056/NEJMoa1714021)
